# Longitudinal dynamic contrast-enhanced MRI radiomic models for early prediction of response to neoadjuvant systemic therapy in triple-negative breast cancer

**DOI:** 10.3389/fonc.2023.1264259

**Published:** 2023-10-24

**Authors:** Bikash Panthi, Rania M. Mohamed, Beatriz E. Adrada, Medine Boge, Rosalind P. Candelaria, Huiqin Chen, Kelly K. Hunt, Lei Huo, Ken-Pin Hwang, Anil Korkut, Deanna L. Lane, Huong C. Le-Petross, Jessica W. T. Leung, Jennifer K. Litton, Sanaz Pashapoor, Frances Perez, Jong Bum Son, Jia Sun, Alastair Thompson, Debu Tripathy, Vicente Valero, Peng Wei, Jason White, Zhan Xu, Wei Yang, Zijian Zhou, Clinton Yam, Gaiane M. Rauch, Jingfei Ma

**Affiliations:** ^1^Department of Imaging Physics, The University of Texas MD Anderson Cancer Center, Houston, TX, United States; ^2^Department of Breast Imaging, The University of Texas MD Anderson Cancer Center, Houston, TX, United States; ^3^Koc University Hospital, Istanbul, Türkiye; ^4^Department of Biostatistics, The University of Texas MD Anderson Cancer Center, Houston, TX, United States; ^5^Department of Breast Surgical Oncology, The University of Texas MD Anderson Cancer Center, Houston, TX, United States; ^6^Department of Pathology, The University of Texas MD Anderson Cancer Center, Houston, TX, United States; ^7^Department of Bioinformatics and Computational Biology, The University of Texas MD Anderson Cancer Center, Houston, TX, United States; ^8^Department of Breast Medical Oncology, The University of Texas MD Anderson Cancer Center, Houston, TX, United States; ^9^Department of Surgery, Baylor College of Medicine, Houston, TX, United States; ^10^Department of Abdominal Imaging, The University of Texas MD Anderson Cancer Center, Houston, TX, United States

**Keywords:** triple-negative breast cancer, dynamic contrast-enhanced MRI, neoadjuvant systemic therapy, radiomic analysis, pathologic complete response

## Abstract

Early prediction of neoadjuvant systemic therapy (NAST) response for triple-negative breast cancer (TNBC) patients could help oncologists select individualized treatment and avoid toxic effects associated with ineffective therapy in patients unlikely to achieve pathologic complete response (pCR). The objective of this study is to evaluate the performance of radiomic features of the peritumoral and tumoral regions from dynamic contrast-enhanced magnetic resonance imaging (DCE-MRI) acquired at different time points of NAST for early treatment response prediction in TNBC. This study included 163 Stage I-III patients with TNBC undergoing NAST as part of a prospective clinical trial (NCT02276443). Peritumoral and tumoral regions of interest were segmented on DCE images at baseline (BL) and after two (C2) and four (C4) cycles of NAST. Ten first-order (FO) radiomic features and 300 gray-level-co-occurrence matrix (GLCM) features were calculated. Area under the receiver operating characteristic curve (AUC) and Wilcoxon rank sum test were used to determine the most predictive features. Multivariate logistic regression models were used for performance assessment. Pearson correlation was used to assess intrareader and interreader variability. Seventy-eight patients (48%) had pCR (52 training, 26 testing), and 85 (52%) had non-pCR (57 training, 28 testing). Forty-six radiomic features had AUC at least 0.70, and 13 multivariate models had AUC at least 0.75 for training and testing sets. The Pearson correlation showed significant correlation between readers. In conclusion, Radiomic features from DCE-MRI are useful for differentiating pCR and non-pCR. Similarly, predictive radiomic models based on these features can improve early noninvasive treatment response prediction in TNBC patients undergoing NAST.

## 1 Introduction

Triple-negative breast cancer (TNBC) accounts for approximately 10% to 20% of breast cancers but almost 30% to 40% of breast cancer–related deaths (1). TNBC is defined by a profile of negative immunohistochemical staining of the receptors for progesterone, estrogen, and human epidermal growth factor (HER2) and therefore not responsive to endocrine or HER2-targeted therapies (2). In addition, TNBC, when not responsive to chemotherapy, is generally associated with a poor prognosis with a high recurrence rate and a low long-term survival rate (3).

In patients with TNBC, neoadjuvant systemic therapy (NAST) with chemotherapy agents such as anthracyclines, taxanes, and cyclophosphamide, carboplatin plus Food and Drug Administration–approved immunotherapy agents such as pembrolizumab is usually administered before surgery to downstage the tumor (2, 4–8). Patients with a pathologic complete response (pCR) to NAST have favorable long-term overall survival and event-free survival, whereas patients without a pCR to NAST have higher recurrence and mortality rates (9, 10). However, only 50% to 60% of TNBC patients achieve a pCR to NAST. Thus, early prediction of NAST response is crucial to avoid exposure of predicted non-responders to ineffective NAST and unnecessary toxicity of neoadjuvant immunotherapy. Early prediction of NAST response can also help oncologists triage patients to a clinical trial and has the potential to better personalize therapy.

Dynamic contrast-enhanced magnetic resonance imaging (DCE-MRI) and the associated temporal enhancement curves can be used in pharmacokinetic modeling to determine the vascular properties of a tumor, such as the integrity and density of the tumor micro-vessels (11). Volumetric changes along with quantitative and semiquantitative kinetic parameters derived from DCE-MRI have been found to be applicable for breast cancer diagnosis, measurement of breast tumor size, classification of breast tumors, and detection of residual breast cancer after NAST (12–18).

In recent years, several studies have been conducted to assess the role of quantitative radiomic imaging features extracted from MRI images in predicting prognosis and treatment response in patients with breast cancer (19, 20). Wu et al. (21) studied quantitative radiomic features from DCE-MRI extracted before and after one cycle of NAST and demonstrated that these features could be used to develop a clinical biomarker for the prediction of the tumor response to NAST. Other investigators also studied radiomic features from DCE-MRI images for the early prediction of tumor response to NAST (12, 19, 21–24). In addition, radiomic features of breast MRI images have been widely investigated for the noninvasive characterization of tumors (25–28).

Studies suggest that peritumoral features assessed by MRI could provide vital information on the tumor microenvironment, cancer development, chemoresistance, and treatment response (29, 30). While previous studies have shown the potential of radiomic analysis in predicting response to NAST, most of these studies have focused on the tumoral region alone. Furthermore, few studies have explored the performance of radiomic models, specifically in patients with TNBC, and even fewer have evaluated the performance of models based on features extracted from DCE-MRI images obtained at multiple time points during NAST.

In this study, we systematically evaluated the performance of models based on radiomic features of both the peritumoral and tumoral regions from DCE-MRI images at baseline (BL), after two cycles (C2), and after four cycles (C4) of NAST for early prediction of response to NAST in TNBC.

## 2 Materials and methods

### 2.1 Patient population

**Figure 1:**
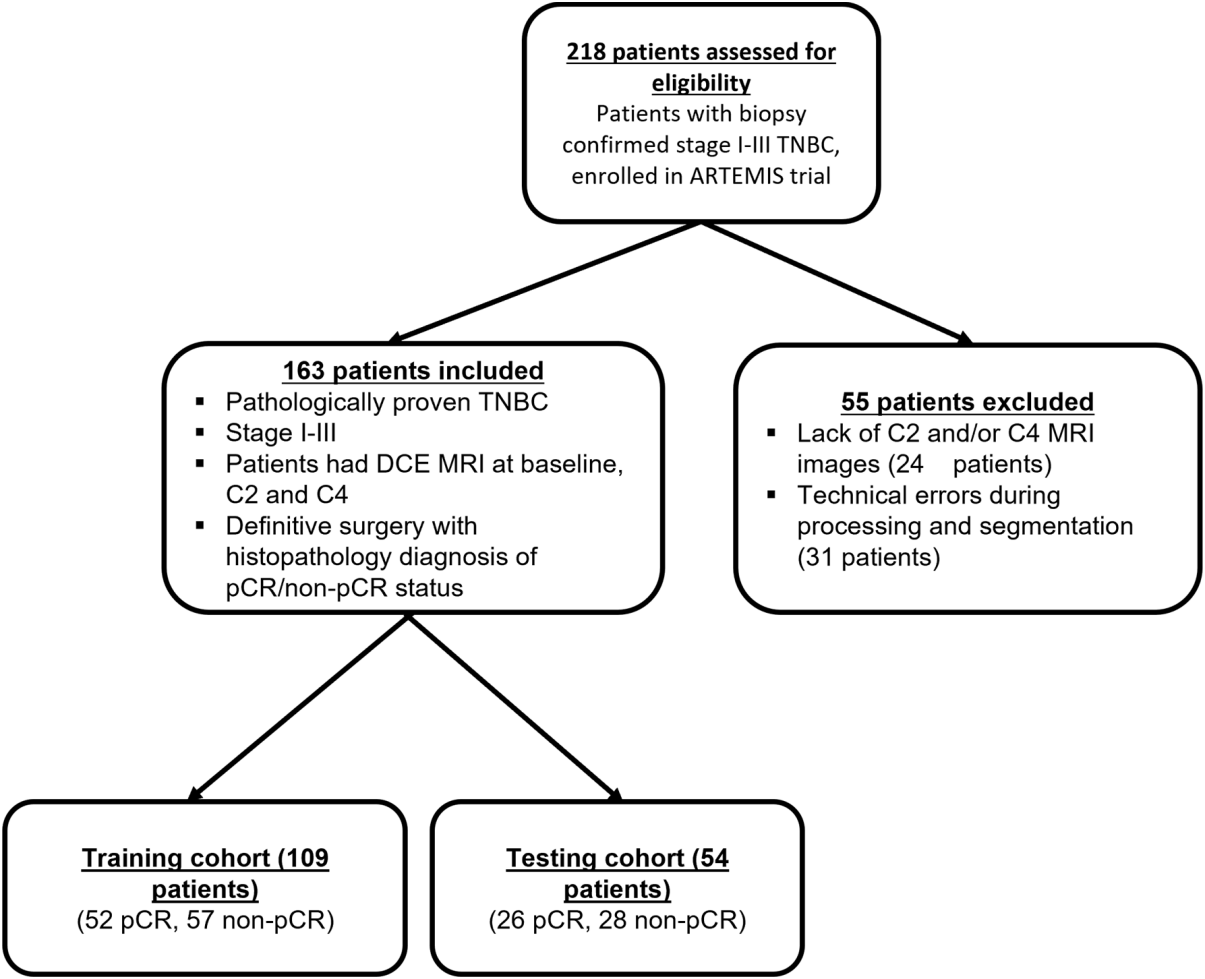
Inclusion and exclusion criteria for the study patients.

In this institutional review board–approved study, we included 163 patients with biopsy-confirmed stage I-III TNBC enrolled in the prospective clinical trial “ARTEMIS: A Robust TNBC Evaluation FraMework to Improve Survival (NCT02276443)”. Informed consent was obtained from all the patients before enrollment in this trial. The inclusion and exclusion criteria are shown in **Figure 1**. TNBC was defined from standard pathologic assays of biopsy specimens as negative for estrogen receptor and progesterone receptor (<10% of tumor staining) and negative for HER2 (immunohistochemistry score < 3, gene copy number not amplified) (31).

All patients in this study received NAST consisting of dose-dense doxorubicin and cyclophosphamide for four cycles, followed by paclitaxel every two weeks for four cycles or weekly for a total of 12 doses. All patients underwent DCE-MRI scans at baseline (BL), after 2 cycles(C2) and after 4 cycles(C4) of NAST, followed by surgery after the completion of NAST. Patient demographic data, clinical information, and pathologic findings were obtained from patients’ medical records. Patients were classified as, having a pCR or a non-pCR according to the findings on the pathologic review of surgical specimens. pCR was defined as the absence of invasive carcinoma in the breast and axillary lymph nodes.

### 2.2 DCE-MRI acquisition

For all the patients, DCE-MRI images were acquired on a GE 3.0 T MR750w whole body scanner (Waukesha, WI) with a bilateral 8-channel phased array coil. The patients were imaged in a prone and feet-in-first position. The imaging protocol included a DCE-MRI series based on the differential subsampling with cartesian ordering (DISCO) sequence. Typical MRI scan parameters used for the DISCO acquisition were as follows: field of view = 34 × 34 cm, slice thickness = 3.0 mm, slice spacing = -1.5 mm, flip angle = 12°, repetition time = 7.6 ms, echo time 1/echo time 2 = 1.1/2.3 ms, total acquisition time = 7 minutes, acquisition matrix = 320 ×320, number of acquired slices = 60-115, temporal resolution = 8-15.5 s, receiver bandwidth = ± 166.7 kHz, and number of excitations = 0.69. During the DCE-MRI acquisition, each patient was injected with a single bolus of gadobutrol (Gadovist, Bayer Health Care) contrast agent (0.1 mL/kg at ~2 mL/s followed by a saline flush) after at least one mask phase was obtained.

### 2.3 Image processing and feature extraction

**Figure 2:**
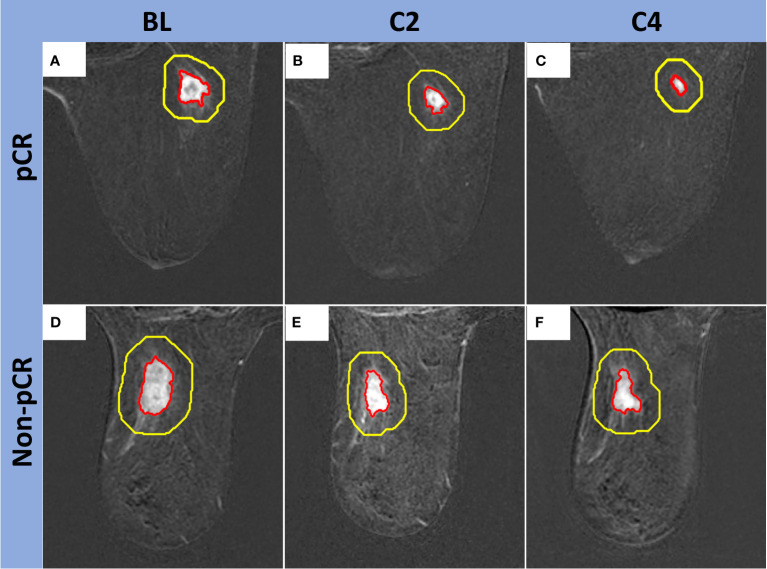
Examples of peritumoral regions automatically generated by the expansion of the tumor ROIs outward. **Top row,** Fifty-seven-year-old woman with a right breast TNBC (red contour) that measured 1.7 x 1.5 x 1.5 cm at BL **(A)**, 1.4 x 1.2 x 0.9 cm at C2 **(B)**, and 1.2 x 0.8 x 0.7 cm at C4 **(C)**. Final surgical pathology showed pCR. **Bottom row,** Seventy-eight-year-old woman with a left breast TNBC (red contour) that measured 2.6 x 2.0 x 1.8 cm at BL **(D)**, 2.8 x 1.5 x 1.4 cm at C2 **(E)**, and 2.2 x 1.7 x 1.3 cm at C4 **(F)**. Final surgical pathology showed non-pCR. Peritumoral segmentations are shown with yellow contours. The area between the yellow and red contours (thickness = 10 mm) was used for peritumoral features measurement.

Manual tumor segmentations, followed by semiautomatic refinement of regions of interest (ROIs), were carried out on 2.5-minute early-phase DCE subtraction images by two fellowship-trained breast radiologists with eight years (MB) and five years (RMM) of experience, respectively. The tumoral region was defined as the region exhibiting contrast enhancement on the DCE images. All the segmentations on BL, C2, and C4 images were performed using an in-house image analysis software program (Image-I v2.0) coded in MATLAB (MathWorks Inc, Natick, MA, USA; RRID: SCR_001622) (31). Additionally, peritumoral regions were automatically generated by expanding the tumor ROIs outward with a fixed thickness of 10 mm (**Figure 2**) (32). A tumor bed was segmented for the cases with no visible tumor enhancement at C2 and/or C4.

**Figure 3:**
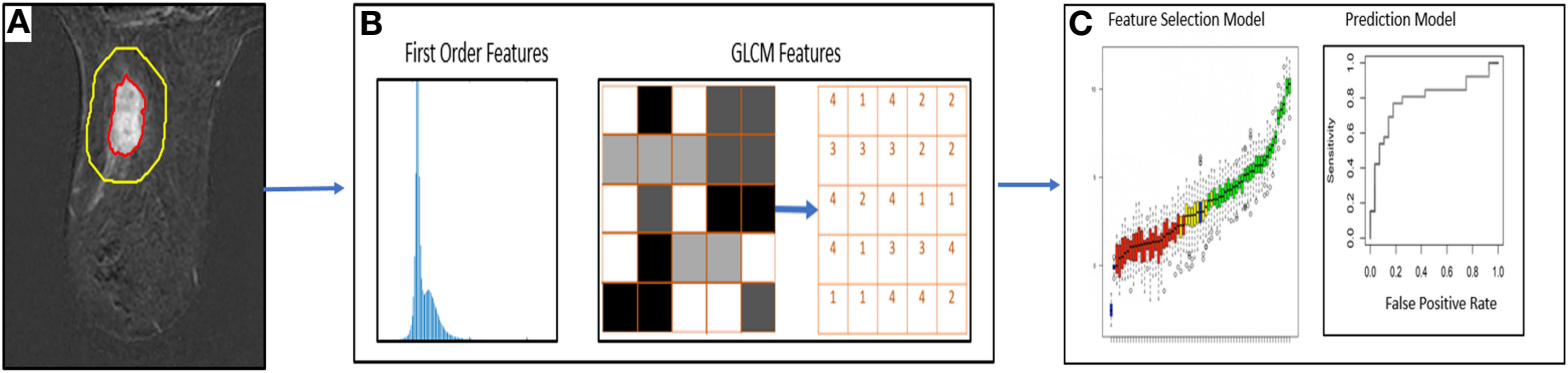
Workflow of DCE-MRI radiomic analysis for prediction of pCR. **(A)** Segmentation of regions of interest in DCE-MRI images **(B)** Extraction of first order radiomic features and GLCM features **(C)** Statistical models for feature selection and prediction of pCR.

A total of 310 radiomic features per imaging time point were extracted separately from the segmented tumor and peritumoral ROIs using an in-house source code based on MATLAB (**Figure 3**). Of the 310 features extracted, 10 were the first order (FO) histogram features (minimum; maximum; mean; standard deviation; 1st, 5th, 95th, and 99th percentiles; skewness; and kurtosis), and the remaining 300 features were gray-level co-occurrence matrix (GLCM) features generated as 60 rotation-invariant features obtained from five different gray levels. For each of the five gray levels (8, 16, 32, 64, and 256), the mean, range, and angular variance of the following 20 GLCM features were calculated to generate the 60 rotation-invariant features: autocorrelation, correlation, contrast, cluster prominence, cluster prominence, cluster shade, dissimilarity, energy, homogeneity, maximum probability, sum of squares/variance, sum average, sum variance, sum entropy, difference variance, difference entropy, information measure of correlation 1, information measure of correlation 2, inverse difference normalized, and inverse difference moment normalized (33).

### 2.4 Statistical analysis

The absolute differences (ADs) and the relative differences (RDs) of radiomic features between the three imaging time points (C4 vs. BL [C4BL], C4 vs. C2 [C4C2], and C2 vs. baseline [C2BL]) were calculated. The calculated radiomic features and their differences from peritumoral and tumoral regions were compared between the patients with pCR and non-pCR using the Wilcoxon rank sum test and Fisher’s exact test. The patient cohort was split into a training set (n=109, 67%) and a testing set (n=54, 33%) in a 2:1 ratio.

For the univariate analysis, area under the receiver operating characteristic curve (AUC) was measured for each radiomic feature from peritumoral and tumoral ROIs at all three imaging time points. Furthermore, AUCs were calculated for the ADs and RDs in the features between these time points. For multivariate analysis, logistic regression with elastic net regularization was performed for texture feature selection. The tuning parameter was optimized by using five-fold cross-validation based on the mean cross-validation AUC. Furthermore, independent testing was performed using the testing data set.

A second set of tumor segmentation was produced independently by a fellowship-trained breast radiologist (SP) with 12 years of experience. The same methodology used to extract features from the initial set of ROIs was applied to extract radiomic features from these new ROIs. The Pearson correlation coefficient was calculated to measure the linear relationship of each feature between two data sets. For the Pearson correlation coefficient, a value of 1 indicates a perfect positive correlation, 0 indicates no correlation, and -1 indicates a perfect negative correlation. Interreader differences were evaluated using the Wilcoxon signed-rank test. The ratio of interreader to intrareader variance was assessed to determine the degree of agreement between two sets of measurements. A lower ratio indicates greater agreement between the readers, suggesting that the measurements are reproducible and consistent.

Statistical analyses were conducted using R software (version 4.0.3, R Development Core Team, Vienna, Austria; RRID: SCR_001905). A *p*-value less than 0.05 was considered statistically significant.

## 3 Results

### 3.1 Patient characteristics

Of the 218 TNBC patients from the ARTEMIS trial who were assessed for eligibility, 55 were excluded from this study, leaving 163 TNBC patients in the final cohort (**Figure 1**). Patient characteristics are reported in **Table 1**. Seventy-eight patients (48%) had a pCR, and 85 (52%) had a non-pCR. There were no statistically significant differences in demographic and clinical characteristics between the patients who achieved a pCR and those who did not.

**Table 1:** Clinical and pathologic characteristics of patients with TNBC undergoing NAST included in the study.

Characteristic	Non-pCR (n = 85) (52%)	pCR (n = 78) (48%)	P value
Median age (range), years	50 (31–78)	48 (23-78)	
T category, n (%)			
T1	12 (14.1)	19 (24.4)	
T2	59 (69.4)	53 (67.9)	
T3	13 (15.3)	5 (6.4)	
T4	1 (1.2)	1 (1.3)	
N category, n (%)			0.850
N0	55 (64.7)	53 (68.0)	
N1	19 (22.4)	17 (21.8)	
N2	4 (4.7)	3 (3.8)	
N3	7 (8.2)	5 (6.4)	
Clinical stage, n (%)			0.690
I	11 (12.9)	11 (14.1)	
II	59 (69.4)	55 (70.5)	
III	15 (17.7)	12 (15.4)	
Histologic type, n (%)			0.741
Invasive ductal carcinoma	76 (89.4)	73 (93.6)	
Metaplastic	8 (9.4)	5 (6.4)	
Apocrine	1 (1.2)	0 (0)	
Type of surgery, n (%)			0.575
Breast-conserving surgery	47 (55.3)	49 (62.8)	
Total mastectomy	38 (44.7)	29 (37.2)	

### 3.2 Univariate analysis

Forty-six radiomic features (21 extracted from the peritumoral region and 25 from the tumoral region) achieved statistical significance in predicting the pCR status of a patient and had AUC at least 0.70 for both the training and testing cohorts (**Tables 2**, **3**). Two tumoral radiomic features (RD-C4BL_percentile 5 and RD-C4BL_percentile 1) had AUC greater than 0.80 for both the training and testing cohorts. The AUCs for the 21 significant features from the peritumoral region ranged from 0.70 to 0.82 for the training cohort and 0.70 to 0.77 for the testing cohort (**Table 2**). Similarly, the AUCs for the 25 significant features from the tumoral region ranged from 0.70 to 0.84 for the training cohort and 0.70 to 0.81 for the testing cohort (**Table 3**). None of the 46 features that were significant and had AUC at least 0.70 in both the training and testing cohorts were GLCM features. Additionally, none of the features extracted from BL and C2 had AUC at least 0.70 for both the training and testing cohorts.

**Table 2:** Significant features (AUC ≥ 0.70 for both training and testing sets) extracted from the peritumoral region as identified from univariate analysis.

Feature	Training (n = 109)		Testing (n = 54)	
	AUC	95% CI	AUC	95% CI
C4				
Peritumoral_DCE_C4_FO_Percentile.95	0.80	0.72-0.88	0.71	0.56-0.86
Peritumoral_DCE_C4_FO_Maximum	0.79	0.71-0.88	0.70	0.55-0.85
Peritumoral_DCE_C4_FO_Percentile.99	0.79	0.70-0.88	0.70	0.55-0.85
Peritumoral_DCE_C4_FO_Mean	0.78	0.70-0.87	0.73	0.59-0.87
Peritumoral_DCE_C4_FO_Percentile.5	0.70	0.60-0.80	0.73	0.60-0.87
AD C4BL				
Peritumoral_DCE_AD-C4BL_FO_Percentile.99	0.79	0.71-0.88	0.74	0.61-0.88
Peritumoral_DCE_AD-C4BL_FO_Maximum	0.78	0.69-0.86	0.72	0.58-0.87
Peritumoral_DCE_AD-C4BL_FO_Percentile.95	0.78	0.69-0.86	0.76	0.62-0.90
Peritumoral_DCE_AD-C4BL_FO_Mean	0.76	0.68-0.85	0.77	0.63-0.91
Peritumoral_DCE_AD-C4BL_FO_Percentile.5	0.70	0.61-0.80	0.70	0.56-0.85
RD C2BL				
Peritumoral_DCE_RD-C2BL_FO_Maximum	0.70	0.60-0.80	0.70	0.56-0.85
RD C4BL				
Peritumoral_DCE_RD-C4BL_FO_Percentile.95	0.82	0.74-0.90	0.76	0.63-0.90
Peritumoral_DCE_RD-C4BL_FO_Percentile.99	0.82	0.74-0.90	0.76	0.62-0.89
Peritumoral_DCE_RD-C4BL_FO_Maximum	0.82	0.74-0.90	0.75	0.61-0.89
Peritumoral_DCE_RD-C4BL_FO_Mean	0.81	0.73-0.90	0.77	0.64-0.90
Peritumoral_DCE_RD-C4BL_FO_Standard.Deviation	0.76	0.66-0.85	0.70	0.54-0.84
Peritumoral_DCE_RD-C4BL_FO_Percentile.5	0.75	0.66-0.84	0.70	0.55-0.85
RD C4C2				
Peritumoral_DCE_RD-C4C2_FO_Percentile.95	0.82	0.74-0.90	0.72	0.58-0.86
Peritumoral_DCE_RD-C4C2_FO_Percentile.99	0.79	0.70-0.88	0.72	0.58-0.86
Peritumoral_DCE_RD-C4C2_FO_Mean	0.79	0.70-0.88	0.72	0.58-0.86
Peritumoral_DCE_RD-C4C2_FO_Maximum	0.76	0.66-0.85	0.70	0.55-0.84

**Table 3:** Significant features (AUC ≥ 0.70 for both training and testing sets) extracted from the tumoral region as identified from univariate analysis.

Feature	Training (n = 109)		Testing (n = 54)	
	AUC	95% CI	AUC	95% CI
C4				
Tumor_DCE_C4_FO_Mean	0.81	0.73-0.89	0.72	0.57-0.86
Tumor_DCE_C4_FO_Percentile.5	0.80	0.72-0.88	0.71	0.57-0.86
Tumor_DCE_C4_FO_Percentile.1	0.79	0.70-0.88	0.74	0.60-0.88
Tumor_DCE_C4_FO_Minimum	0.74	0.64-0.83	0.77	0.63-0.90
AD C4BL				
Tumor_DCE_AD-C4BL_FO_Percentile.99	0.80	0.71-0.88	0.73	0.59-0.87
Tumor_DCE_AD-C4BL_FO_Percentile.95	0.79	0.70-0.87	0.76	0.62-0.90
Tumor_DCE_AD-C4BL_FO_Percentile.5	0.79	0.70-0.87	0.81	0.69-0.94
Tumor_DCE_AD-C4BL_FO_Mean	0.79	0.70-0.87	0.77	0.63-0.90
Tumor_DCE_AD-C4BL_FO_Percentile.1	0.78	0.70-0.87	0.81	0.69-0.93
Tumor_DCE_AD-C4BL_FO_Maximum	0.78	0.70-0.87	0.72	0.58-0.87
Tumor_DCE_AD-C4BL_FO_Minimum	0.71	0.61-0.81	0.76	0.62-0.90
AD C4C2				
Tumor_DCE_AD-C4C2_FO_Percentile.1	0.76	0.68-0.85	0.71	0.57-0.85
RD C2BL				
Tumor_DCE_RD-C2BL_FO_Maximum	0.70	0.60-0.80	0.71	0.57-0.85
RD C4BL				
Tumor_DCE_RD-C4BL_FO_Mean	0.84	0.77-0.92	0.78	0.64-0.91
Tumor_DCE_RD-C4BL_FO_Percentile.95	0.83	0.76-0.91	0.75	0.62-0.89
Tumor_DCE_RD-C4BL_FO_Maximum	0.83	0.76-0.91	0.75	0.61-0.89
Tumor_DCE_RD-C4BL_FO_Percentile.99	0.83	0.76-0.91	0.75	0.61-0.89
Tumor_DCE_RD-C4BL_FO_Percentile.5	0.83	0.75-0.90	0.80	0.68-0.93
Tumor_DCE_RD-C4BL_FO_Percentile.1	0.82	0.74-0.90	0.81	0.69-0.93
Tumor_DCE_RD-C4BL_FO_Minimum	0.79	0.70-0.87	0.79	0.65-0.92
RD C4C2				
Tumor_DCE_RD-C4C2_FO_Mean	0.84	0.77-0.92	0.71	0.57-0.85
Tumor_DCE_RD-C4C2_FO_Percentile.5	0.83	0.75-0.90	0.77	0.65-0.90
Tumor_DCE_RD-C4C2_FO_Percentile.1	0.80	0.72-0.89	0.76	0.63-0.89
Tumor_DCE_RD-C4C2_FO_Percentile.99	0.79	0.71-0.88	0.70	0.56-0.85
Tumor_DCE_RD-C4C2_FO_Minimum	0.70	0.59-0.80	0.76	0.62-0.89

### 3.3 Multivariate analysis

Multivariate analysis identified 13 radiomic models with AUC at least 0.75 for the training and testing cohorts (**Table 4**).

**Table 4:** Testing and training AUC for the 13 logistic regression models that achieved AUC at least 0.75 for both training and testing sets using radiomic features from BL, C2, and C4.

Radiomic model	Training (n = 109)		Testing (n = 54)	
	AUC	95% CI	AUC	95% CI
Peritumoral_DCE_AD-C4BL_FO	0.79	0.71-0.88	0.76	0.62-0.90
Peritumoral_DCE_RD-C4BL_FO	0.88	0.82-0.94	0.76	0.63-0.90
Peritumoral_DCE_BL_C2_C4_FO	0.95	0.92-0.99	0.76	0.62-0.90
Peritumoral_DCE_BL_C2_C4_AD-C2BL_RD-C2BL_AD-C4BL_RD-C4BL_AD-C4C2_RD-C4C2_FO	0.95	0.91-0.98	0.79	0.65-0.92
Tumoral_DCE_AD-C4BL_FO	0.82	0.74-0.90	0.76	0.63-0.90
Tumoral_DCE_RD-C4BL_FO	0.87	0.80-0.93	0.78	0.64-0.91
Tumoral_DCE_BL_C4_AD-C4BL_RD-C4BL_FO	0.88	0.81-0.94	0.75	0.61-0.90
Peritumoral_Tumoral_DCE_AD-C4BL_FO	0.79	0.71-0.88	0.76	0.62-0.89
Peritumoral_Tumoral_DCE_RD-C4BL_FO	0.89	0.84-0.95	0.77	0.64-0.91
Peritumoral_Tumoral_DCE_BL_C4_AD-C4BL_RD-C4BL_FO	0.91	0.86-0.96	0.75	0.61-0.90
Peritumoral_Tumoral_DCE_C2_C4_AD-C4C2_RD-C4C2_FO	0.93	0.88-0.98	0.75	0.61-0.89
Peritumoral_Tumoral_DCE_BL_C2_C4_FO	0.97	0.95-0.99	0.76	0.62-0.90
Peritumoral_Tumoral_DCE_BL_C2_C4_AD-C2BL_RD-C2BL_AD-C4BL_RD-C4BL_AD-C4C2_RD-C4C2_FO	0.96	0.93-0.99	0.78	0.65-0.91

**Figure 4:**
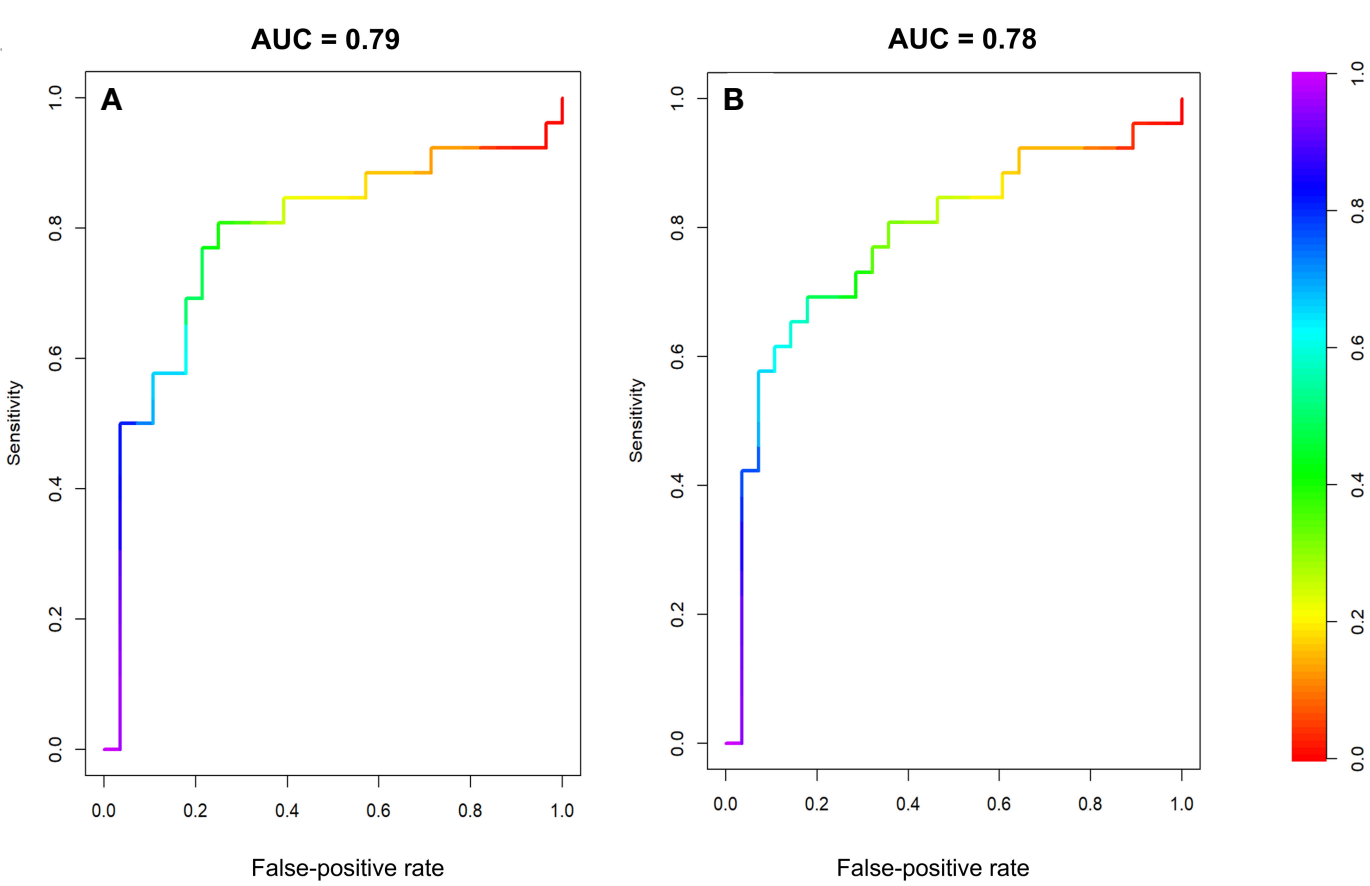
Receiver operator characteristic curves for the testing datasets of two multivariate models. **(A)** Model 1, using FO features from the peritumoral region at BL, C2, and C4 and the differences between these time points. **(B)** Model 2, using FO features from both the peritumoral and tumoral regions BL, C2, and C4 and the differences between these time points.

The multivariate radiomic models with the best AUCs in the testing cohort were based on peritumoral FO features acquired at BL, C2, and C4 along with their ADs and RDs (model 1: AUC in the training cohort, 0.95 [95% confidence interval (CI): 0.91-0.98], AUC in the testing cohort, 0.79 [95% CI: 0.65-0.92]) and a model based on features from both the peritumoral and tumoral regions (model 2: AUC in the training cohort, 0.96 [95% CI: 0.93-0.99], AUC in the testing cohort, 0.78 [95% CI: 0.65-0.91]). The receiver operative characteristic curves for model 1 and model 2 are shown in **Figure 4**.

The radiomic models based on peritumoral features at BL only, C2 only, a combination of BL and C2, as well as the models based on tumoral features at BL only, a combination of C2 and C4, had AUCs less than 0.70 for testing cohorts. However, the radiomic models based on peritumoral features at C4 only, a combination of BL and C4, a combination of C2 and C4, as well as tumoral features at C2 only, C4 only, a combination of BL and C2, and a combination of BL and C4 had AUCs greater than 0.70 for both training and testing cohorts (**Table 5**). The radiomic model based on tumoral features from BL, C2 and C4 had an AUC of 0.99 [95% CI: 0.99-1.00] in the training cohort and an AUC of 0.73 [95% CI: 0.59-0.87] in the testing cohort. The radiomic models based on peritumoral features from BL, C2 and C4 with/without tumoral features from BL, C2, and C4 had AUCs greater than 0.75 for both training and testing cohort (**Table 4**).

**Table 5:** Logistic regression models that achieved AUC > 0.70 for both training and testing sets using first order radiomic features from BL, C2, and C4.

Radiomic model	Training (n = 109)		Testing (n = 54)	
	AUC	95% CI	AUC	95% CI
Peritumoral_DCE_C4_FO	0.86	0.79-0.93	0.71	0.57-0.85
Peritumoral_DCE_BL_C4_FO	0.90	0.84-0.96	0.73	0.59-0.87
Peritumoral_DCE_C2_C4_FO	0.93	0.88-0.97	0.74	0.60-0.88
Tumoral_DCE_C2_FO	0.87	0.80-0.94	0.71	0.56-0.85
Tumoral_DCE_C4_FO	0.81	0.73-0.89	0.71	0.57-0.85
Tumoral_DCE_BL_C2_ FO	0.81	0.73-0.89	0.72	0.58-0.86
Tumoral_DCE_BL_C4_FO	0.89	0.83-0.95	0.74	0.61-0.88

### 3.4 Interreader variability analysis

The Pearson correlation analysis showed that both GLCM and FO features exhibited strong interreader correlation. Specifically, the correlation coefficient was greater than 0.8 for 83% (25/30) of original FO features, 60% (18/30) of AD FO features, and 67% (20/30) of RD FO features. For GLCM, the correlation coefficient was greater than 0.8 for 100% (300/300) of original and AD GLCM features and 90% (271/300) of RD GLCM features. For the ratio of interreader to intrareader variance for the FO features, the mean was 5.6 × 10 ^-3^ and the median was zero, indicating high agreement between the interreader datasets. The standard deviation was 0.02, indicating a slight variation in agreement between the readers across FO features. The range was relatively narrow, 0 to 0.08, suggesting that most features had a high level of agreement between the readers. There was no variability in the measurements between the readers for any of the GLCM features. The ratios were all zero, indicating a high level of agreement and consistency between the readers.

## 4 Discussion

In this study, we used longitudinal DCE-MRI images obtained before the start of doxorubicin and cyclophosphamide-based NAST and after two and four cycles of NAST to assess radiomic features from the peritumoral and tumoral regions for early prediction of NAST response in TNBC patients. In the univariate analysis, we identified 46 radiomic features able to predict pCR with an AUC at least 0.70 for both the training and testing cohorts. Furthermore, in the multivariate analysis, we found that 13 multivariate radiomic models had AUC at least 0.75 for both the training and testing cohorts for early prediction of NAST response in TNBC.

Our results revealed that FO radiomic features from DCE-MRI were better predictors of treatment response than GLCM features. None of the 300 GLCM features had AUC at least 0.70 for both the training and testing cohorts; thus, their usefulness in predicting NAST response could not be established. The interreader variability analysis showed high reliability and reproducibility with a slightly better interreader agreement in GLCM features than in FO features. GLCM features capture texture information of the image, which is less subjective and more reproducible across readers than the FO features, which are mostly visual features. Furthermore, the features extracted from baseline DCE-MRI images and the models using features only from baseline DCE-MRI images showed poor performance in terms of AUC. Similar to this finding, Panthi et al. have previously reported that the tumor size measurements (longest dimension, tumor volume, enhanced tumor volume and functional tumor volume) extracted from DCE-MRI images at baseline showed poor performance. The tumor measurements extracted at C2, C4, and their relative differences (C2 vs BL and C4 vs BL) showed good correlation with the treatment response with a maximum AUC of 0.84 [95% CI: 0.76-0.92] for functional tumor volume at C4 (31). The possible explanation of these findings is that tumor biology can evolve over the course of treatment. The radiomic features measured after the initiation of NAST may capture these changes in tumor biology better than features measured at baseline, contributing to the better predictive performance of the features measured after NAST initiation.

In our study, almost 50% (6/13) of the radiomic models with AUC at least 0.75 for both the training and testing cohorts were based on a combination of peritumoral and tumoral features. The inclusion of the peritumoral features along with the tumoral features may have improved the performance of radiomic models by capturing the infiltrative tumor margins and information about the tumor microenvironment. Additionally, peritumoral features have the potential to aid in predicting treatment response, as tumor behavior heterogeneity likely reflects variation both of the tumor and surrounding environment.

MRI-based radiomic analysis has shown promise in predicting treatment response in breast cancer (12, 19, 22, 23, 34). Cain et al. used pretreatment tumor features from 151 patients with TNBC and HER2-enriched breast cancer in a multivariate machine learning model and showed an AUC of 0.707 for pCR prediction (35). In a study including 83 breast cancer patients, Pesapane et al. extracted 136 representative radiomic features of the tumoral region from pretreatment T1-weighted contrast-enhanced MRI images to predict the response to NAST. Their radiomic model had an AUC of 0.64 (95% CI, 0.51-0.75), which increased to 0.83 (95% CI, 0.73-0.92) after the investigators combined the radiomic model with clinical and biological features (36). Similarly, Fan et al. performed a radiomic analysis of pretreatment DCE-MRI images in 57 patients and demonstrated that combining features from the tumoral region and background parenchyma significantly improved the performance of their radiomic model (19). Li et al, in a study of 33 patients, compared AUCs for pretreatment images and images obtained after one cycle of NAST and observed better predictive AUCs after one cycle of treatment (37). In contrast to the aforementioned studies, we created multivariate radiomic models based on DCE-MRI images at three imaging time points to extract features from peritumoral as well as tumoral regions in a much larger cohort limited to patients with TNBC.

Braman et al. conducted a study including 117 breast cancer patients (with hormone-receptor-positive, HER2-negative, triple-negative, and HER2-positive disease) and showed that combined peritumoral and tumoral radiomic features obtained at baseline could be utilized to predict pCR to neoadjuvant chemotherapy (22). These authors reported an AUC of 0.74 for the testing cohort, which was slightly lower than the AUC of 0.79 for the testing cohort in our study, which had a larger patient pool, was limited to patients with TNBC, and was based on radiomic features obtained at multiple time points. Caballo et al. (38) used a four-dimensional machine learning radiomics approach to assess breast cancer response to NAST. They extracted 348 features from peritumoral and tumoral regions from DCE-MRI images to develop predictive radiomic models. These authors reported a multivariate model with an AUC of 0.71 for the data set of 251 patients (107 with luminal A subtype, 47 with luminal B subtype, 25 with HER2-enriched subtype, and 72 with TNBC). Furthermore, their study showed that the predictive performance improved when the radiomic models were tailored to specific subtypes of breast cancer. Caballo et al. found an AUC of 0.80 in 72 TNBC patients (38), which is comparable to the testing-group AUC of 0.79 in our study of 163 TNBC patients.

Fan et al. reported a study in which clinical features and radiomic features from the tumoral region before and after two cycles of treatment were acquired for 114 patients with primary breast cancer. These authors observed an AUC of 0.57 for the models based on pretreatment features and an AUC of 0.77 for the model based on posttreatment features. The combined model based on pretreatment features, posttreatment features, relative net feature change, Jacobian maps, and clinical features had an AUC of 0.81 (39). Our findings are consistent with their findings in that our findings showed improvement in the predictive performance with a combined model with features from BL, C2, C4, and their net feature changes.

Our study has several limitations. First, our study was conducted on a single scanner platform at a single institution, and therefore our findings will need to be validated on other scanner platforms and at different institutions. Second, although our study is the largest radiomic analysis reported to date for treatment response prediction in TNBC patients, additional validation may be necessary in a larger study with better statistical power and was only tested on patients with preoperative AC chemotherapy. Finally, the peritumoral regions were uniformly defined with a fixed dilation of 10 mm around the tumor ROIs for all patients, regardless of tumor size. Future studies with different dilation radii based on individual tumor properties can offer a more comprehensive understanding of the role of the peritumoral region in response prediction.

## 5 Conclusions

In summary, our study shows that radiomic models based on peritumoral and tumoral features from longitudinal DCE-MRI images were able to serve as noninvasive biomarkers for early prediction of NAST response in patients with early-stage TNBC. Radiomic features extracted after four cycles of treatment and their change relative to baseline were better predictors of response than those from baseline only. Further, FO radiomic features showed better predictive performance than GLCM features.

## Data availability statement

The datasets presented in this article are not readily available because we are not allowed to share patient data. Requests to access the datasets should be directed to jma@mdanderson.org.

## Ethics statement

The studies involving humans were approved by The UT MD Anderson Cancer Center IRB. The studies were conducted in accordance with the local legislation and institutional requirements. Written informed consent for participation in this study was provided by the participants’ legal guardians/next of kin.

## Author contributions

BP: Conceptualization, Formal Analysis, Methodology, Writing – original draft. RMM: Methodology, Writing – review & editing. BA: Data curation, Methodology, Writing – review & editing. MB: Formal Analysis, Methodology, Writing – review & editing. RC: Data curation, Methodology, Writing – review & editing. HC: Formal Analysis, Writing – review & editing. KKH: Writing – review & editing. LH: Writing – review & editing. K-PH: Writing – review & editing. AK: Writing – review & editing. DL: Writing – review & editing. HL-P: Writing – review & editing. JWTL: Writing – review & editing. JKL: Writing – review & editing. SP: Formal Analysis, Methodology, Writing – review & editing. FP: Writing – review & editing. JBS: Formal Analysis, Methodology, Writing – review & editing. JS: Formal Analysis, Writing – review & editing. AT: Writing – review & editing. DT: Writing – review & editing. VV: Writing – review & editing. PW: Writing – review & editing. JW: Writing – review & editing. ZX: Formal Analysis, Methodology, Writing – review & editing. WY: Writing – review & editing. ZZ: Formal Analysis, Methodology, Writing – review & editing. CY: Writing – review & editing. GR: Conceptualization, Formal Analysis, Methodology, Supervision, Writing – review & editing. JM: Formal Analysis, Methodology, Supervision, Writing – review & editing.
